# Combining Slaughterhouse Surveillance Data with Cattle Tracing Scheme and Environmental Data to Quantify Environmental Risk Factors for Liver Fluke in Cattle

**DOI:** 10.3389/fvets.2017.00065

**Published:** 2017-05-08

**Authors:** Giles T. Innocent, Lucy Gilbert, Edward O. Jones, James E. McLeod, George Gunn, Iain J. McKendrick, Steve D. Albon

**Affiliations:** ^1^Biomathematics and Statistics Scotland, JCMB, Edinburgh, UK; ^2^The James Hutton Institute, Aberdeen, UK; ^3^Department of Infectious Disease Epidemiology, London School of Hygiene and Tropical Medicine, London, UK; ^4^Future Farming Systems, R&D Division, SRUC, An Lòchran – Inverness Campus, Inverness, UK

**Keywords:** liver fluke, *Fasciola hepatica*, fasciolosis, *Galba truncatula*, cattle, slaughterhouse, environment, risk factors

## Abstract

Liver fluke infection causes serious disease (fasciolosis) in cattle and sheep in many regions of the world, resulting in production losses and additional economic consequences due to condemnation of the liver at slaughter. Liver fluke depends on mud snails as an intermediate host and infect livestock when ingested through grazing. Therefore, environmental factors play important roles in infection risk and climate change is likely to modify this. Here, we demonstrate how slaughterhouse data can be integrated with other data, including animal movement and climate variables to identify environmental risk factors for liver fluke in cattle in Scotland. We fitted a generalized linear mixed model to the data, with exposure-weighted random and fixed effects, an approach which takes into account the amount of time cattle spent at different locations, exposed to different levels of risk. This enabled us to identify an increased risk of liver fluke with increased animal age, rainfall, and temperature and for farms located further to the West, in excess of the risk associated with a warmer, wetter climate. This model explained 45% of the variability in liver fluke between farms, suggesting that the unexplained 55% was due to factors not included in the model, such as differences in on-farm management and presence of wet habitats. This approach demonstrates the value of statistically integrating routinely recorded slaughterhouse data with other pre-existing data, creating a powerful approach to quantify disease risks in production animals. Furthermore, this approach can be used to better quantify the impact of projected climate change on liver fluke risk for future studies.

## Introduction

Liver fluke, *Fasciola hepatica*, is a trematode flatworm parasite which causes serious disease (fasciolosis) in livestock, especially cattle and sheep, in many regions of the world. It is of widespread importance as it is a generalist parasite infecting not only cattle and sheep but also any mammal that ingests infective cysts, including pigs, donkeys (1), deer, rabbits, hares (2, 3), kangaroos (4), and even humans (5, 6). Liver fluke infection imposes health and welfare costs on the animal, such as weight loss, anemia, and reduced productivity [e.g., Ref. (7, 8)] and, for some animals such as sheep, it can cause death (9, 10). Fasciolosis prevalence is often high, even over large geographic regions such as Western Europe. For example, in adult dairy cattle, recent fluke prevalence estimates have been reported to be between 72 and 80% in the UK (11, 12), 61% in Spain, 50% in Germany, and 37% in Belgium (13–15). Liver fluke thus generates serious economic costs for livestock producers (16–18). For example, it has been estimated that liver fluke costs the UK dairy and beef industries approximately £23 million each year (19).

*Fasciola hepatica* has several life stages and requires an intermediate host: in the UK, this is most commonly the mud snail *Galba truncatula* (formerly *Lymnaea truncatula*) [see Ref. (10) for more detailed life cycle]. Fluke eggs excreted in livestock feces hatch out to release the first life stage, tiny miracidia that infect mud snails in which they develop into the next stage, cercaria. These cercariae are released from the snail where they swim until they encyst on vegetation as metacercariae, the infective stage (20). In cyst form, they are robust and can survive even in cool or dry conditions until ingested by grazing livestock where they emerge in the small intestine and migrate across the gut wall and into the liver. The juvenile flukes feed and move through the liver, causing destruction and hemorrhage of the liver tissue before eventual migration to the bile ducts where the flukes mature and lay eggs. This process causes illness or death in the infected animal and damage to the liver resulting in the liver being condemned at the slaughterhouse; approximately 30% of cattle livers were condemned in (rejected by) slaughterhouses in 2011–2012 in Scotland at standard meat inspection due to the damage caused by liver fluke (10).

Fasciolosis is endemic in the UK and is also considered an emerging disease since the incidence of infection and geographic distribution has increased greatly in the last two decades (10). As well as factors such as the creation of wetland areas for the conservation of wading birds (21), a key reason for this increase is thought to be climate change, especially a warmer and wetter climate (12, 21–25).

Climate and local variation in weather between years both influence liver fluke infection because *F. hepatica* spends much of its life outside the host in the environment, with the intermediate host (mud snail) and the free-living stages of *F. hepatica* both requiring wet conditions. The influence of environmental factors on the risk of liver fluke infection has long been known: the first model of liver fluke risk with respect to weather conditions was developed in 1959 (26) and used to predict years when infection will be more prevalent. More recent modeling work relating liver fluke infection to environmental factors indicated that geography and climate data together may explain 70–76% of variation in fluke infection prevalence (27), while Howell et al. (12) found that rainfall alone explained 24% of variation in liver fluke between dairy herds.

To detect liver fluke infection and to provide estimates of infection prevalence, most studies use fecal egg counts [e.g., Ref. (28–30)] or the detection of antibodies in blood serum samples [e.g., Ref. (28, 30, 31)] or in bulk milk samples [e.g., Ref. (12, 28)]. These approaches all require dedicated sample collection and laboratory work. Some studies have used liver condemnation at slaughter as the outcome of interest [e.g., Ref. (32, 33)]. However, to the authors’ knowledge, no studies have used the complete life history of animals to attempt to determine the risk associated with each farm in that chain.

Slaughterhouses in the UK routinely record whether or not livers are condemned due to fasciolosis. Although not usually recorded, if each liver record is linked to an identified animal, because the UK has a cattle tracing scheme, data can be accessed that specify the period that each animal spent at every holding unit throughout its life. Our aim is to trial the use of slaughterhouse data on condemned livers as a novel approach to model the environmental risk factors influencing liver fluke infection. To do this, we combine liver data sourced from a single slaughterhouse in Scotland with each animal’s movement history (from the cattle tracing scheme) together with existing GIS-based environmental data for each location, using a novel variant of a generalized linear mixed model.

We aim to demonstrate that, using this modeling approach, slaughterhouse surveillance data, when linked to cattle tracing administrative data, can be used to quantify the environmental risk factors for liver fluke infection at the level of the individual animal. This has implications for future predictions of liver fluke risk at the regional or national scale due to climate change, as well as defining a methodology to facilitate further use of slaughterhouse surveillance data.

## Materials and Methods

### Slaughterhouse Data

We used data from 7,858 cattle slaughtered at J. M. Munroe’s abattoir in Dingwall, Scotland, between December 2007 and December 2009. Data included animal identification, whether or not the liver was condemned by the Meat Hygiene Service (MHS) inspector, age (all less than 30 months old), and sex. The recording and transcription of the assessment of the liver by the MHS inspector and the linkage of these data to the eartag number for the animal were done on a voluntary basis by arrangement with the individual staff concerned. These data would not normally have been recorded.

### Cattle Life Histories

**Figure 1:**
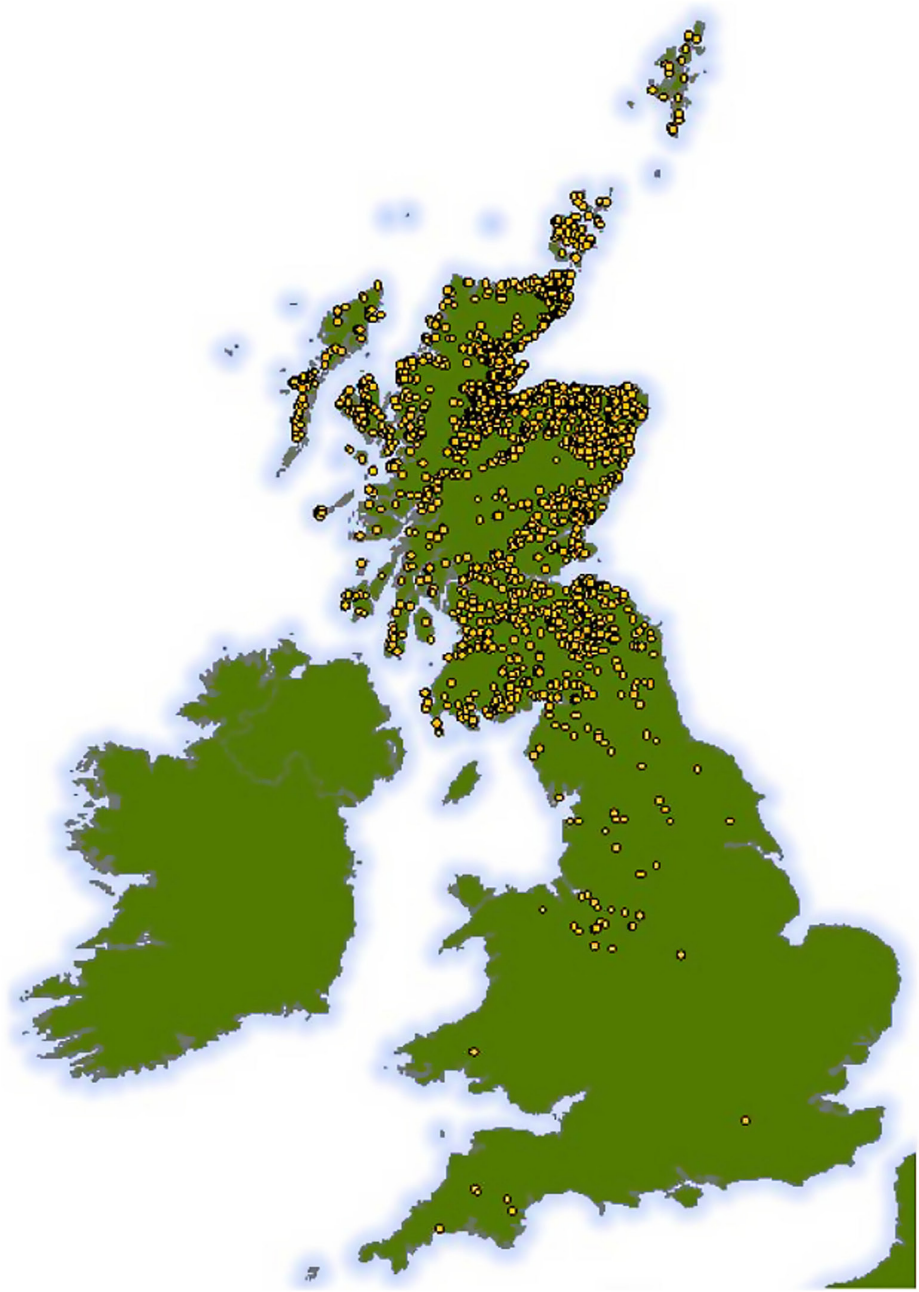
**Map showing the geographic range of all 2,068 holding units at which the 7,858 cattle spent time before slaughter**. Approximate locations only are shown, as all positions have been plotted with the addition of some random spatial noise to preserve anonymity while ensuring that the map still represents the broad distribution of cattle holding units used in our study across the UK.

Using the cattle identification and the UK cattle movement database (cattle tracing scheme), the movement history for each animal was identified, locating where, when, and for how long it had stayed on different farms. The cattle tracing scheme was set up in the UK in 1989, subsequent to the bovine spongiform encephalopathy epidemic to ensure that all cattle entering the food chain could be traced from their farm of birth through to slaughter. Since 2001, all movements are recorded in an electronic database. In total, 2,078 premises were involved in the raising of these animals, of which 2,068 unique addresses were identified (Figure 1). The number of farms on which a single animal had been reared varied from one to six. We estimated the total number of “risk days” during which each animal was potentially at risk of infection on each farm. Most cattle in Scotland are housed in barns during the winter but are out on pastures for the rest of the year; even where cattle are not housed indoors, low winter temperatures result in minimal levels of fluke transmission. Relatively low numbers of the intermediate snail host will be active, and those snails present will have low numbers of cercariae (34). Accordingly, we defined “risk days” as the number of days between 1st April and the 30th September that an animal spent at each location. We acknowledge that this assumption is a potential source of error in the model, because of variations in cattle housing periods and winter temperatures. However, this assumption does not affect our aim of trialing the use of slaughterhouse data in a novel modeling approach.

### Environmental Data

Using data in a Geographic Information System [ArcMap; (35)], we extracted environmental variables associated with each farm location on a digital map (36). The locations of the cattle were assumed to be within 2 km of the farm location so a 2-km radius circle was generated around each point location using the ArcMap (35) *buffer* function. The Hawth’s Tools (37) *Zonal Statistics* tool was used to calculate the mean value (within each circle) for the raster maps for temperature and rainfall parameters. These parameters were the annual average of the daily mean temperature (in degrees Celsius) and the annual average of the daily rainfall (in millimeters). Again, while this assumption is a potential source of error, since a minority of farms may keep cattle outside this zone, any errors in temperature and rainfall covariates will be small compared to inter-regional or across-Scotland differences in climate.

### Statistical Model to Assess Risk Factors

The data were statistically challenging because of the need to identify environmental risk factors for liver fluke when the animals have moved around the country spending periods of time in different locations with different environmental and geographical characteristics. We therefore developed a variant of a generalized linear mixed model to estimate the effect on the probability of liver condemnation at an individual level of different risk factors, some of which operate at the farm level, with these latter effects being linearly weighted by how long each animal spent, at risk, on each farm. This approach allowed us to model multiple potential risk factors (age, sex, location, temperature, and rainfall) as fixed and random effects, at both the individual animal and farm levels.

If the ingestion of fluke metacercaria is thought of as a non-homogeneous Poisson process, and subsequent development into an adult fluke, and observation at slaughter are treated as random Bernoulli events, the total number of fluke observable within an animal’s liver at any point in time would be expected to be a random variable following a Poisson distribution where λ, the mean number of fluke, is an integral of the historic instantaneous risk over the animal’s lifetime, scaled in some way for non-development and non-observation. Indeed, almost all parasite burden count data do exhibit a Poisson distribution (38). From the properties of the Poisson distribution, the probability of observing no fluke is therefore e^−λ^. Such an animal would be recorded as not having its liver condemned. A model of this type was first formulated by Fisher (39). We assume that λ increases with all the animal-associated risk factors and all the daily risks encountered by an animal throughout its life. The way that the various risk factors over the life time of animal affect the probability of its liver being condemned at slaughter can be expressed mathematically as follows:

$$Y_{i}=\{\begin{array}{l} \text{0 with probability }e^{-\lambda_{i}}\\ \text{1 with probability }1-e^{-\lambda_{i}}. \end{array}$$

*Y_i_* is a random variate which is the outcome of a Bernoulli trial (condemnation of the liver) with probability of “success” $p_{i}=1-e^{-\lambda_{i}}$, where

$$\text{ln}(\lambda_{i})=\sum_{j}\alpha_{j}x_{ij}+\sum_{l,k}\beta_{l}W_{ik}f_{kl}+\sum_{k,m}\gamma_{km}W_{ik}$$

and ln denotes the natural logarithm (log to the base *e*); α*_j_* is the fixed effect coefficient associated with the *j*th animal-level covariate for animal *i*; *x_ij_* is the *j*th animal-level covariate for animal *i*; β*_l_* is the fixed effect coefficient associated with the *l*th farm-level covariate; *W_ik_* is a weight which depends upon the length of time that animal *i* spent on farm *k, W_ik_* ≡ 0 when animal *i* has spent no time on farm *k*; *f_kl_* is the *l*th farm-level covariate for farm *k*; and γ*_km_* is the *m*th random effect for farm *k*.

The observation of condemned or non-condemned livers provides sufficient information for us to make inference about the parameters contributing to the means λ*_i_* across the population of animals.

We can use the above mathematical model of liver condemnation to produce a linear relationship between the observed outcome (condemnation or non-condemnation of the liver) and explanatory variables as follows:

$$\sum_{j}(\alpha_{j}x_{ij})+\sum_{l,k}(\beta_{l}W_{ik}f_{kl})+\sum_{k,m}(\gamma_{km}W_{ik})=\text{ln}(-\text{ln}(1-p_{i})).$$

This is similar to a standard generalized linear model with complementary-log–log link function (40) but with the crucial difference that this model weights the farm-level effects (both fixed and random effects) by the number of risk days that the animal spent on each farm. Hence, parameters $\beta_{l}$ and γ*_m_* are estimated as rates, since they enter the model as products with the numbers of days at risk. It is assumed that the risk associated with farm-level covariates accrues linearly with risk days. In addition, importantly, this model allows an animal not only to be influenced by different covariates but also to be affected by different realizations of a covariate during its lifetime, as it moves from farm to farm. This allows us to make inference across the whole of an animal’s lifetime, resulting in less uncertainty in farm-based fixed and random effects.

In our model, the response variable was whether or not the liver was condemned due to liver fluke. The model can be thought of as apportioning the risk of condemnation of a liver into risk factors specific to an animal (animal-level fixed effects) and risk attributable to those farms on which the animal has spent time (farm-level fixed and random effects).

The fixed effects examined were, at the animal level, those associated with age and sex and, at the farm level, those associated with rainfall in millimeters (the average daily rainfall at the farm site), temperature in degrees (the average daily temperature at the farm site), and geographic location as kilometers North and kilometers East of the Ordnance Survey grid origin point. Farm covariates were weighted by the estimated number of days at risk. Variables were deemed non-significant if they had a 95% credible interval that included 0, and such fixed effects were removed from the initial model. An exception to this protocol was that if either the easting or northing was significant, both would be kept in the model. The fixed effect of farm location accounts for linear spatial effects. Since North and East are arbitrary directions, both are retained in the model to account for any first-order spatial effect that does not exactly align with either direction.

The farm-level random effects measure risk associated with unmeasured local area effects such as region-specific farming practices or contagion (estimated by the spatially smooth random effect) and unmeasured farm-specific effects such as farm-specific housing periods, habitat, or drainage (this is the site-specific residual risk). These effects are modeled as being independent of one another. The geographical locations of all farms were used to fit a smooth random field, modeling how risk might vary across space; the field interacted with the statistical model *via* the random realization of the field at each farm location. In addition, farm identification number was fitted as a discrete random effect to represent any potential unrecorded farm management effects. Essentially, these are residual farm-level random effects, fitted once all other farm-level fixed and spatially smooth random effects have been estimated. The consistent handling of farm locations in the spatially smooth random field gives us more confidence that these residual random effects will be able to account for localized farm-level variability whether associated with any unrecorded environmental factors or site-specific farming practices. In Section “Results,” each part of the model is considered separately.

The generalized linear mixed model was fitted using integrated, nested Laplace approximation (INLA) using the R-INLA package in R (41–44). The spatially smooth random effect of farm location was fitted using the “spde” model. A triangular mesh was defined on the area containing the observations. This mesh includes data points (locations of the farms) as vertices; in consequence, the resulting realizations at each farm location have consistent statistical properties. Formally, a Gaussian Markov random field was fitted, with stochastic partial differential equations used to incorporate spatial correlation between points. Within the framework of the overall model, the realizations of the field at farm locations are spatially smooth random effects. A more complete description of this method and of the form taken by the spatially smooth surface can be found in Ref. (45).

The ability of the final model to describe the variability seen in the data was evaluated by comparing the hyper-parameter in the final model that captured the variability in the residual, discrete farm-level random effects with the equivalent hyper-parameter in a null model (a baseline model where only an intercept and the residual, discrete farm-level random effects were estimated). The hyper-parameter in this case is analogous to the variance component associated with a random effect in a generalized linear mixed model. It is not possible to estimate excess variability at the lowest stratum of the model (the individual animal) because the response at this stratum is binary (liver condemned or not condemned). Hence, the farm-specific residual effect summarizes any excess of variability in the data relative to the model. Therefore, a comparison of the hyper-parameters that describe these random effects in the final and null models is equivalent to comparing the residual variance of a model to the total variance seen in the data.

## Results

**Figure 2:**
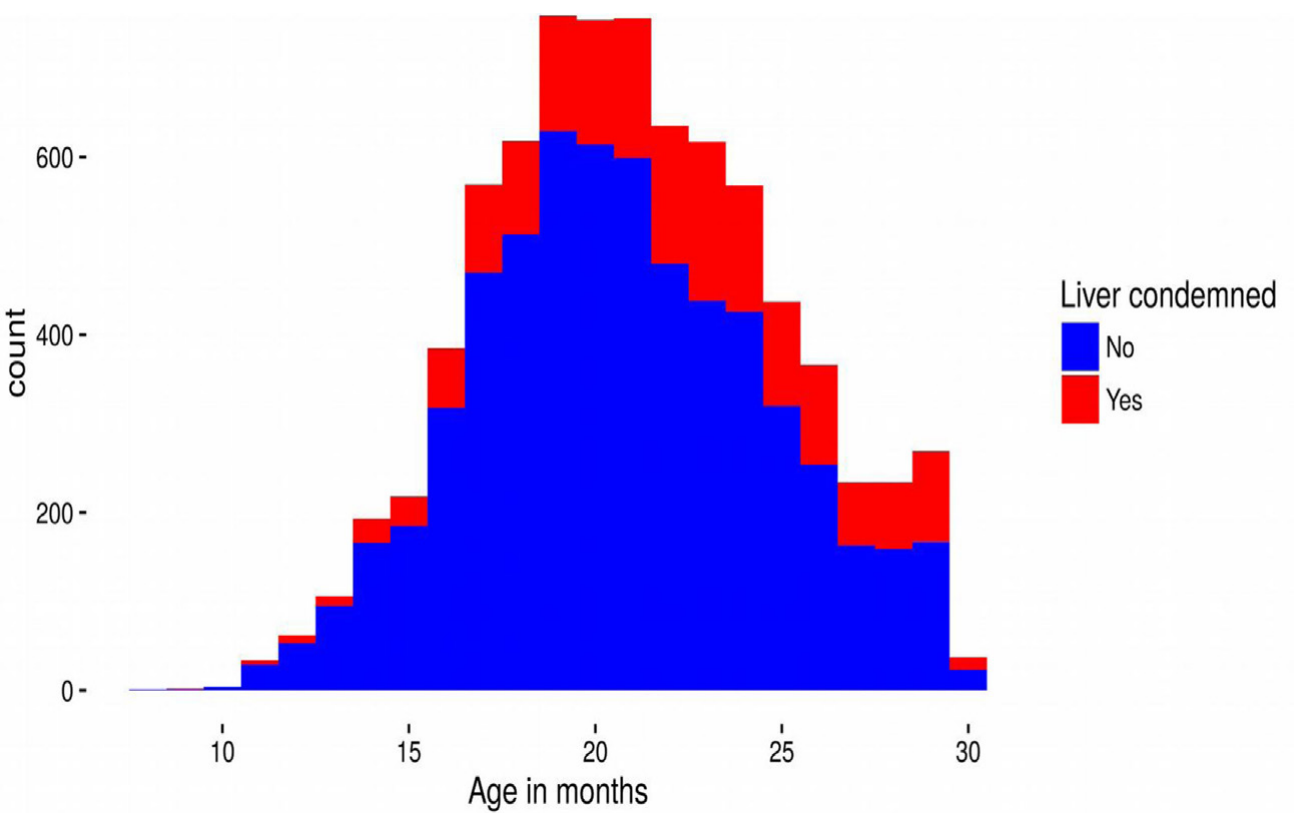
**Stacked frequency distribution of the numbers of cattle livers that were condemned (red) and not condemned (blue) due to liver fluke as a function of age (in months) at slaughter**. The plot summarizes the raw data unadjusted by the model.

Cattle ages in this study ranged from 9 to 30 months old and 4,798 (61%) animals were male. Although all the cattle in our dataset were slaughtered in Dingwall, they had spent periods of time at locations over much of Scotland, and even some locations in England (Figure 1). Of the 7,858 cattle livers recorded, 1,751 (22%) were condemned due to liver fluke infection. However, the prevalence varied with age of the animal increasing steadily from 15% infection in cattle less than 12 months old to over 35% at 30 months (Figure 2).

For simplification of presentation, we describe the three classes of effect (fixed, spatial random, and non-spatial random) separately, but it should be stressed that the model fits them simultaneously, and hence, all estimated effects are conditioned on the rest of the model. Therefore, for example, we should think of the spatially smooth random effect as describing the influences which make neighboring farms similar, once the model has accounted for farm-level fixed effects and site-specific residual effects.

The final fitted model, including fixed effects, and random field and discrete spatial random effects explained 45% of the observed variability in liver condemnation probabilities between farms.

### Fixed Effects

Prevalence in females was 25.2% and in males was 20.4%, but after adjusting for other factors, the 95% credible interval for the difference included 0, and sex was therefore removed from the final model. The final model indicated a significantly higher probability of liver fluke-associated liver condemnation for animals spending more time at risk in areas that had higher average annual rainfall, had warmer climates, were further West, and for older animals (Table 1).

**Table 1:** **Estimates of fixed effects in the final fitted model of liver condemnations due to liver fluke**.

Covariate	Mean value	Median value	95% credible interval
**Age of animal** (days)	0.0026	0.0026	**0.0017–0.0035**
**Easting^a^** (1,000 km)	−0.0293	−0.0292	**−0.0360 to −0.0292**
Northing^a^ (1,000 km)	0.0002	0.0001	−0.0028 to 0.0032
**Rainfall^a^ (**mm)	0.0011	0.0011	**0.0008–0.0015**
**Temperature^a^ (**°)	0.0003	0.0003	**0.0001–0.0006**

*Text in bold reports effects where the 95% credible interval does not include 0*.

*^a^All farm-level effects are weighted by (multiplied by) the animal’s number of at risk days for which it was present on that farm. Therefore, the coefficients should be considered as per day, for example the coefficient associated with Easting should be considered as per day at risk per 1,000 km*.

**Figure 3:**
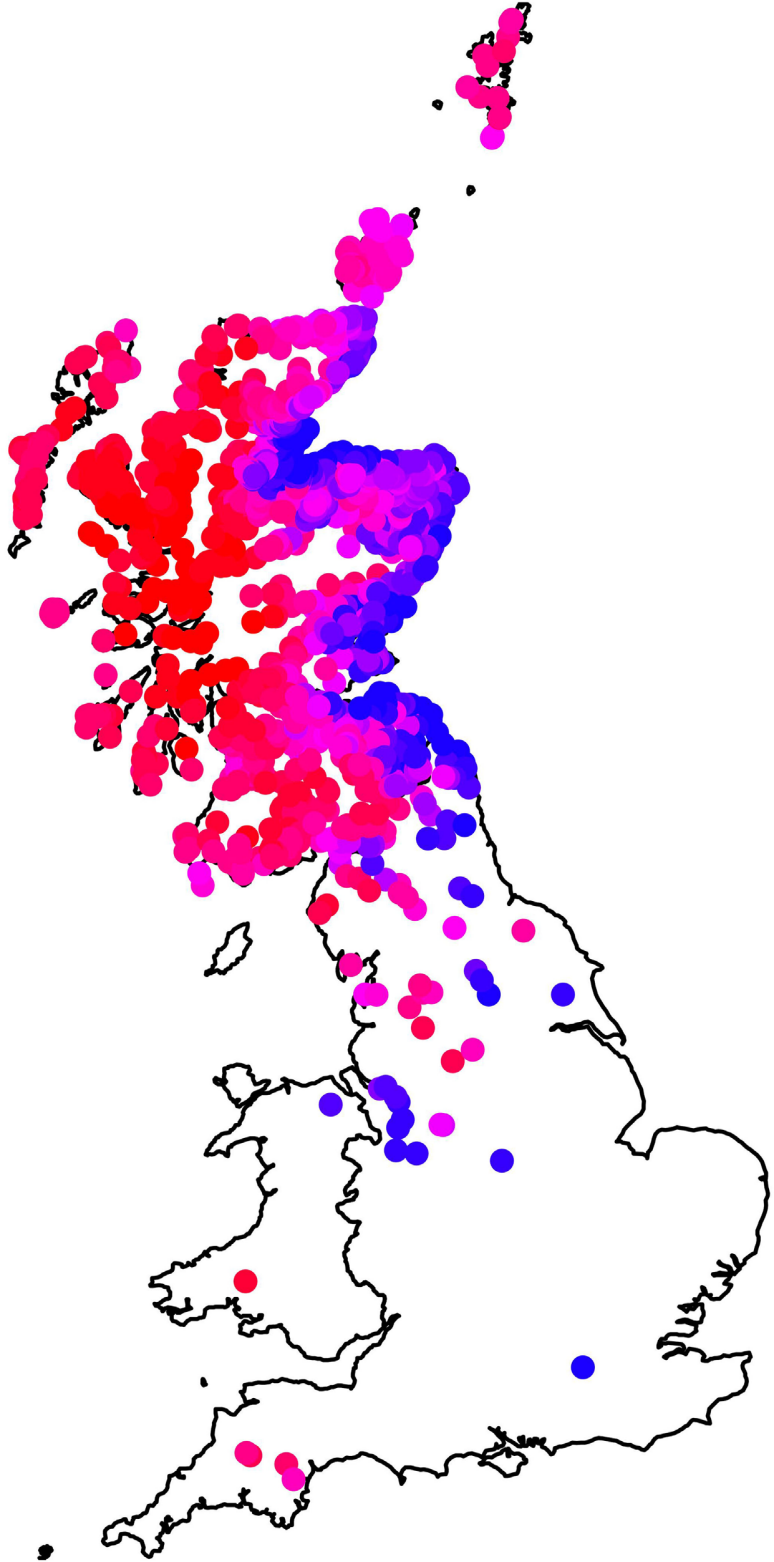
**Map showing the influence of farm-level fixed effects (temperature, rainfall, northings, and eastings) in the model by plotting the mean value, summing across these effects, for each farm in the data set**. Farms plotted in red have the highest risk, those in blue the lowest risk, and farms in pink (high–medium) and purple (medium–low) intermediate risk.

Figure 3 summarizes the summed effect of all the farm-level fixed effects in the model (East–West and South–North clines, and using long-term average rainfall and long-term average temperature) on the estimated mean on-farm risk. In general, farms in the East have lower risk than those on the West, but the risk in the East is particularly low at the coast. The latter property is associated with the effect of local temperature and rainfall. The larger scale East–West effect is statistically significant even after taking into account climatic variables, indicating the influence of further, unobserved, geographic-associated factors on higher fluke risks in the West.

### Spatially Smooth Random Effect

**Figure 4:**
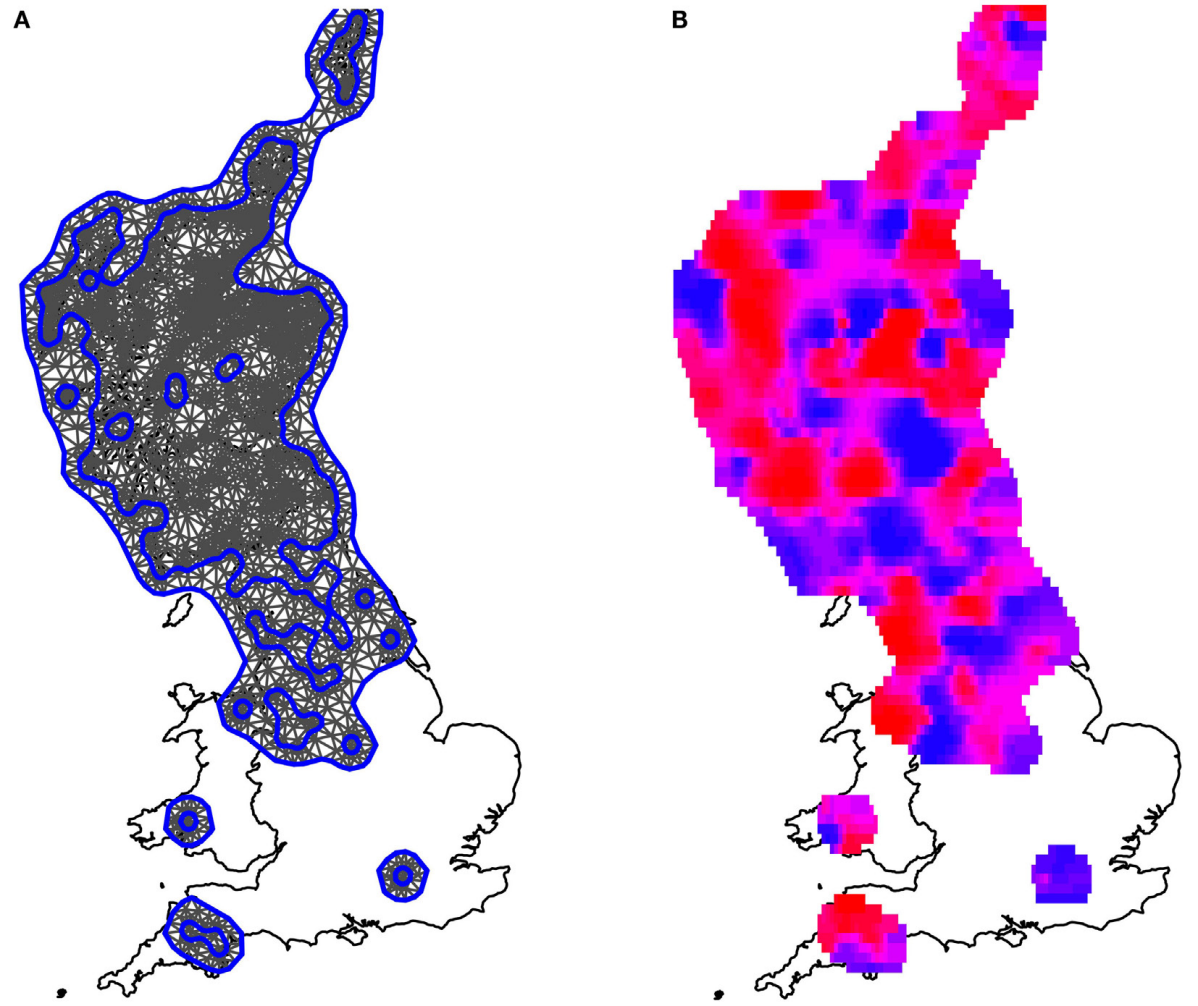
**Maps illustrating the spatially smooth random effect**. **(A)** The mesh used to fit the model, which included the observed farms as vertices. Blue lines are construction lines used by the algorithm in creating the mesh appropriately. **(B)** The mean local risk surface inferred by the model, plotted across the areas for which data were available. Areas shaded in red are areas of high local risk. Areas shaded in blue are areas of low local risk. Although variations can be seen, these fluctuations are small when compared to the uncertainty associated with the estimates; there is no evidence that any areas have either statistically significantly higher or lower localized risks than average, once spatial clines and local climate have been fitted to the model.

The mesh used to fit the data to the model is presented in Figure 4A. After allowing for fixed effects (rainfall, temperature, northings, and eastings), the model spatially smooth random effect generated the mean daily risk rates plotted in Figure 4B. The associated standard errors, however, were high, such that all spatial estimates had a 95% credible interval that included 0 (i.e., there is no strong statistical evidence of any localized spatial effects beyond those described by the East–West cline and local climate as shown in Figure 3). The spatially smooth field was kept in the model, however, to ensure that the site-specific residual effect contained no element attributable to regional location, since in areas with few farms the power to formally detect such an effect is small.

### Site-Specific Residual Risk

**Figure 5:**
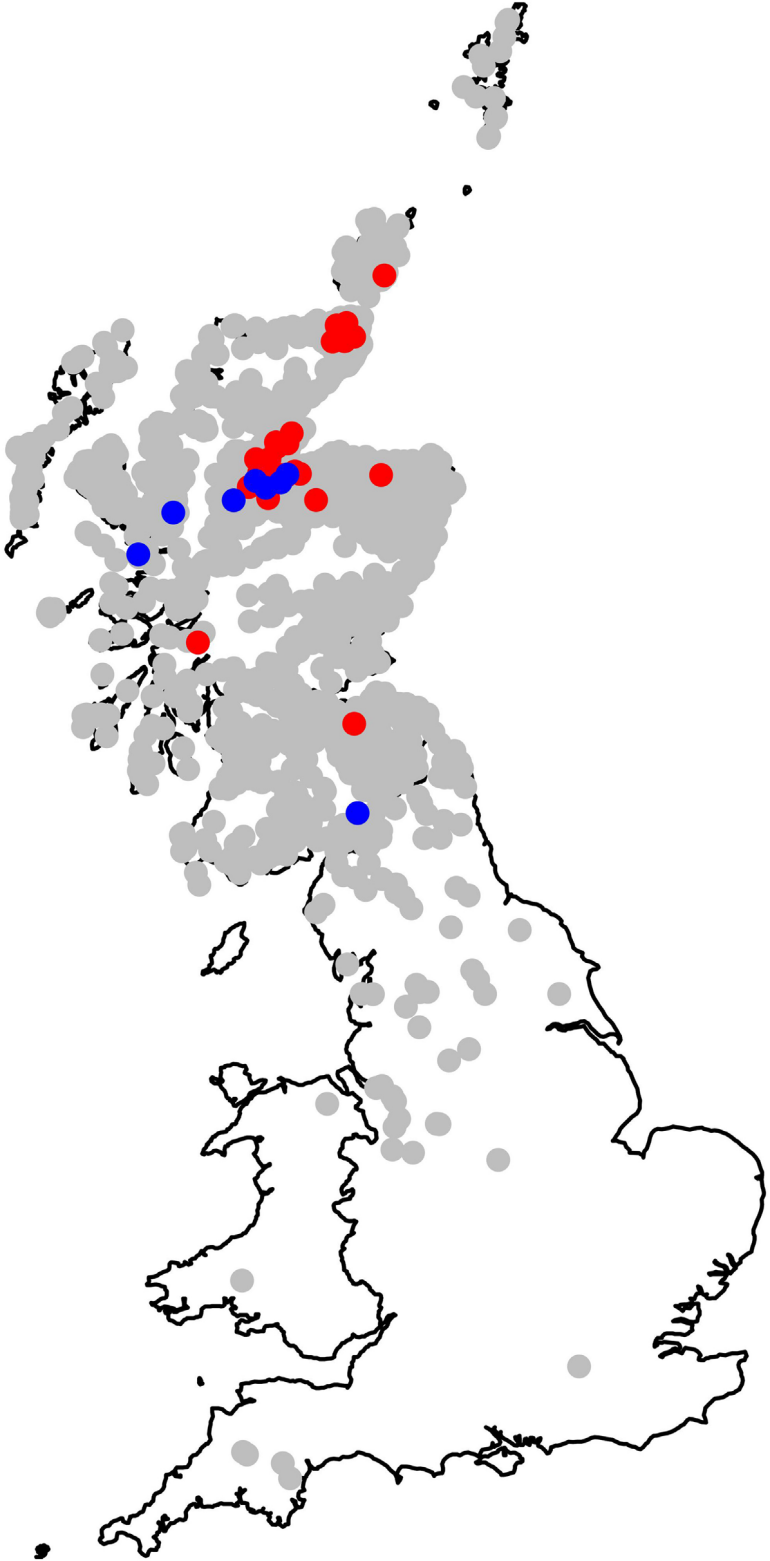
**Map showing the estimated site-specific residual risk for each of the modeled cattle holding units**. Positions plotted in gray are those where the 95% credible interval includes 0; the risk at these farms does not significantly differ from the average. Positions plotted in red are those where the 95% credible interval is entirely greater than 0 (higher risk than the average farm), and those in blue are where the 95% credible interval is entirely less than 0 (lower risk than the average farm). This represents the residual risk attributable to each farm once spatial clines (northings and eastings), local climate (rainfall and temperature), and local similarities between farms (modeled *via* the spatially smooth random effect) have been fitted to the model.

Figure 5 summarizes the estimated site-specific residual effect (unobserved farm effects such as farm-specific housing periods, habitat, micro-climate, or drainage) once the spatially smooth random effect and all fixed effects have been fitted. Figure 5 indicates that most farms had residual liver fluke daily risk rates that did not differ significantly from the average (0). The map shows, however, the approximate location of farms where daily risk rates were higher than expected and those where the risk was lower than expected, given all other factors in the model. The relatively extreme high or low risk at these farms is, therefore, most likely to be due to farm-specific effects such as housing, local management (drainage, fencing off wet areas, etc.), or the presence of habitat that affects snail populations.

## Discussion

By combining, for the first time, existing data on (i) condemned livers from a Scottish slaughterhouse, (ii) cattle movements, and (iii) climate, we have developed a model that identifies the risk factors for liver fluke infection in cattle. This is novel in using slaughterhouse surveillance data and in developing a statistical model which takes into account all the locations at which an animal had spent time. This approach indicates a higher probability of infection in the West of Scotland, independent of this part of the country being warmer and wetter. There is generally lower risk in the East of Scotland, where it is drier and cooler due to a weaker oceanic influence. As well as climatic and geographic risk factors, we also found a higher probability of liver fluke infection in older cattle. This is to be expected as, the longer an animal lives, on average, the more risk of exposure it is likely to have had, and any fluke in the liver will have had more time to cause visible damage, leading to liver condemnation in the slaughterhouse. These are similar to risk factors identified in previous studies that used different modeling approaches and different data (from a variety of regions), e.g., fecal egg counts or antibodies in serum or bulk milk [e.g., Ref. (12, 26, 27, 46, 47)], suggesting that the use of data on condemned livers through slaughterhouse surveillance is robust and applicable to other regions and livestock systems. Other authors using slaughterhouse records (32, 33) found spatial differences in farm risk as well as an association with wet areas.

Exploring different aspects of spatial patterns in the data, a geographical covariate (the East–West cline) was found to be statistically significant as a fixed effect in explaining pattern in the data. After allowing for these high-level trends, the smooth random field showed no evidence for localized regional differences. However, at the finest spatial scale, there was some evidence of individual farms having higher or lower probabilities of infection than average.

Since liver fluke risk is higher in warmer and wetter areas, the warmer and wetter climate predicted for Scotland by current climate change models is likely to increase the severity of the risk and to expand the range of areas that are most at risk across Scotland [and other countries where studies have identified similar risk factors, e.g., England, Wales, and Belgium (12, 26, 27, 46, 47)]. Our modeling approach, by quantifying the effect of climatic variables on fluke risk, has potential to develop predictive models and generate quantitative risk maps for liver fluke under different climate change projections.

Our model, including the effects of cattle age, climate, animal history, and geographic location explained 45% of the variation in observed risk of liver fluke condemnations in animals at slaughter between farms. The variation in liver fluke condemnation risk that remained unexplained was 55% and may be largely due to unrecorded on-farm effects that influence the risk of liver fluke infection (12, 46, 48). Previous studies have identified such on-farm risk factors as the presence of snail habitat (wet areas) on pastures, herd size, length of the grazing season, and proportion of grazed grass in the diet (31, 46, 49). A model by Howell et al. (12), using data on antibodies in bulk milk samples, indicated that such on-farm factors explained 21% of the observed variation in liver fluke infection between dairy herds in Scotland. There are many potential reasons why studies differ in their estimates of the contributions to liver fluke risk of such on-farm effects. Apart from differences in what constitutes an “on-farm effect” (e.g., management practices versus local habitat and climate), there may also be systematic disparity in quantitative outputs between studies because of differences between dairy cattle and cattle sold for beef, and in differences in the response variable used: liver condemnation compared to milk antibody level or fecal egg count.

There are both advantages and disadvantages in our approach for identifying liver fluke risk factors. The slaughterhouse surveillance data provided to us represent a relatively minor increase in effort by the meat inspectors and required no dedicated sampling or laboratory work; also, data are recorded in a direct fashion at the level of the individual animal, thus increasing the power available for the analysis. However, this information was supplied by MHS inspectors on a voluntary basis, based on a local arrangement. Other authors have highlighted the potential for using slaughterhouse data for surveillance of a number of conditions and for syndromic surveillance [e.g., Ref. (50–52)]. It is possible that, as our model takes into account the whole life history of each animal, it increases the amount of information available, thereby facilitating a more powerful analysis. A particular strength of this approach is that, by using the complete history of an animal in a carefully parameterized generalized linear mixed model, we are able to examine the risk on farms that do not directly sell animals to slaughter. This ability to highlight farms with abnormal condemnation risk is one of the important outcomes from this approach and could be used to target follow-up on-farm investigations. In addition, the use of condemned livers avoids some potential issues that can arise from other liver fluke indicators; for example, data derived from fecal counts can be biased by mistaken observation of rumen fluke *Calicophoron daubneyi* (53) and antibody analysis of bulk milk samples do not provide information at the individual animal level. Condemned liver data do not have such issues.

However, there are also negative aspects to our approach. For example, farm management practices that mitigate liver fluke risk by restricting the grazing period are particularly problematic for our model, because the model assumes a standardized period at pasture, so the effect of such strategies will affect the model in a non-random fashion. Such farm-based interventions might explain some of the extreme, farm-specific random effects observed in the model (Figure 5). Our model is also limited by using data from only one slaughterhouse. Although the locations at which cattle spent time were scattered broadly across Scotland and even parts of England, the majority of locations (and time spent at locations) were in NE Scotland, around the region of the slaughterhouse. While the NE of Scotland is one of the main cattle-producing parts of Scotland, the use of one slaughterhouse does result in other areas of the country being relatively under-represented in our data. While our model provided risk factors similar to those from other studies, more accurate and robust outputs could be obtained by using existing liver data from a larger number of slaughterhouses around Scotland. However, our aim of trialing the use of slaughterhouse surveillance data for identifying liver fluke risk factors (made possible by the use of a bespoke statistical model) was, nonetheless, achieved successfully.

In conclusion, we have demonstrated that the use of existing individual animal-level data from slaughterhouse surveillance identifies the same environmental/climatic/geographic risk factors as previous studies from different regions. This result implies that these slaughterhouse liver data are robust enough for use in monitoring liver fluke over time and over space (when coupled with cattle tracing scheme data) and could be used for projecting future fasciolosis risk given likely climate changes or other changes in the environment and pattern of animal movements or management systems. Predictive hazard maps, such as the ones we have produced here, can help in the development of mitigation strategies; for example, a potential strategy to slow the development of resistance to antihelminthic drugs (54) could be to restrict their use to high-risk areas as identified by models such as ours that use slaughterhouse condemnation data. The inclusion of detailed on-farm management factors data and, especially, mud snail data, would undoubtedly increase the predictive power of models of liver fluke infection, but the use of innovative statistical models to combine information from existing datasets does offer a cost-effective way to generate the evidence base for policy interventions. This work highlights how slaughterhouse surveillance data can be a powerful research tool for identifying prevalence and risk factors for disease.

## Ethics Statement

The study entailed recording of the normal meat inspection process of animals sent to slaughter for human consumption. No ethical approval was required nor sought as no alteration was required to the normal processing of these animals.

## Author Contributions

GI: design of analysis, performed analysis, and prepared manuscript. LG: consideration of impact of environment and prepared manuscript. EJ: transcribed data, initial data exploration and analysis, and critical revision of manuscript. JM: preparation of environmental data and critical revision of manuscript. GG: initial negotiations with meat inspectors, advice on technicalities of data, and critical revision of manuscript. IM: design of analysis, statistical input at several points, and critical revision of manuscript. SA: initial data exploration, providing context for geographical data, and critical revision of manuscript. All authors have given final approval to the manuscript.

## Conflict of Interest Statement

The authors declare that the research was conducted in the absence of any commercial or financial relationships that could be construed as a potential conflict of interest.
